# Prevalence of Hypovitaminosis D in Adults Presenting With Generalized Aches: A Hospital-Based Observational Study

**DOI:** 10.7759/cureus.106564

**Published:** 2026-04-07

**Authors:** Bidhan Neupane, Prajwal Kafle, Abashesh Bhandari, Prayush Sharma

**Affiliations:** 1 Internal Medicine/Rheumatology, Nepal Medical College and Teaching Hospital, Kathmandu, NPL; 2 Internal Medicine, Nepal Medical College and Teaching Hospital, Kathmandu, NPL; 3 Internal Medicine, Nepal Medical College, Kathmandu, NPL; 4 Rheumatology, Kathmandu Institute of Science and Technology (KIST) Medical College and Teaching Hospital, Lalitpur, NPL

**Keywords:** generalized ache, hypovitaminosis d, overlap, rheumatic disease, widespread pain

## Abstract

Introduction and aim: Generalized aches mean widespread musculoskeletal pain. It has various etiologies, and among them, hypovitaminosis D is one of the common causes. This study aimed to determine the prevalence of hypovitaminosis D and to identify other causes of generalized aches in adults.

Methods: This retrospective, observational, descriptive study was conducted at Nepal Medical College and Teaching Hospital (NMCTH), Kathmandu, Nepal. Records of adults presenting to the rheumatology OPD with generalized aches from January 1, 2023, to December 31, 2024, were reviewed. Serum 25(OH)D and other relevant investigations were assessed. Patients with coexisting hypovitaminosis D and rheumatic disease were categorized as having hypovitaminosis D-rheumatic disease overlap. Rheumatic causes of widespread pain, other than hypovitaminosis D, were classified as rheumatic disease. Results were expressed as numbers, percentages, and means.

Results: Among 136 patients with generalized aches, the female-to-male ratio was 9.4:1, and 73 (53.7%) had hypovitaminosis D (40 isolated and 33 overlapping rheumatic disease) with a mean vitamin D level of 18.40 ng/mL. The mean age of subjects with hypovitaminosis D was 50.37 years, with female predominance (48.5%). The remaining 63 (46.3%) cases had rheumatic disease with normal levels of vitamin D as a cause of widespread pain, with benign joint hypermobility syndrome (BJHS) being the most common.

Conclusions: Hypovitaminosis D was a common cause of generalized aches in adults and can overlap with rheumatic conditions. Among rheumatic diseases, BJHS was more prevalent.

## Introduction

Generalized aches mean widespread/global musculoskeletal pain. It can lead to disability and impaired quality of life [1]. The reported etiologies for this condition can be grouped into infection (mainly viral), hormonal (thyroid disorder, diabetes mellitus), rheumatic/musculoskeletal disorders (rheumatoid arthritis {RA}, spondyloarthropathies {SpA}, systemic lupus erythematosus {SLE}, fibromyalgia, benign joint hypermobility syndrome {BJHS}), malignancy (multiple myeloma, bone metastasis), and psychiatric (somatoform disorders, depression) [2].

Vitamin D deficiency is one of the most common causes of generalized aches [3]. Low vitamin D levels may lead to decreased bone mass, bone pain, fractures, muscle weakness, and falls. So, the patients with hypovitaminosis D often complain of widespread/global musculoskeletal pain [4]. It has become a major public health concern [5].

Vitamin D is a steroid hormone that exists mainly in two forms: vitamin D2 and vitamin D3 [6]. It is essential for bone formation, maintenance, and remodeling, as well as muscle function. Vitamin D insufficiency or deficiency on the basis of 25-hydroxy vitamin D (25{OH}D) level is still a matter of discussion. Yet serum 25(OH)D >30 ng/mL, 20-30 ng/mL, and <20 ng/mL are considered optimal, insufficient, and deficient, respectively [7].

According to a study, around one billion people globally have hypovitaminosis D [8]. Available studies have reported a prevalence of hypovitaminosis D ranging from 57% to 84.5% in Nepal [9,10]. According to these studies, 36-92% were vitamin D insufficient, and 8-63% were vitamin D deficient [11-13].

Nowadays, many patients are complaining of widespread pain. There are various sources responsible for this condition. Among them, vitamin D deficiency is one of the most common causes. So, the attending physician has been prescribing vitamin D supplements to those patients without performing a test. This practice in our country has led to overlooking other etiologies of generalized aching, as well as to inadequate data on hypovitaminosis D. Therefore, this study was conducted to determine the prevalence of hypovitaminosis D and to identify other causes of generalized aches, including rheumatic diseases in adults, with or without overlap.

## Materials and methods

This retrospective, observational, descriptive study was conducted at Nepal Medical College and Teaching Hospital (NMCTH), Kathmandu, Nepal. Records of the patients who presented to the rheumatology OPD from January 1, 2023, to December 31, 2024, were considered. Both male and female subjects ≥18 years of age with generalized aches were included. Cases of patients aged <18 years were excluded. Generalized aches were defined as widespread/global musculoskeletal pain, which can lead to disability and impaired quality of life [1].

Each patient with generalized aches was evaluated with a thorough history and clinical examination. Serum 25(OH)D, complete blood count (CBC), erythrocyte sedimentation rate (ESR), C-reactive protein (CRP), random blood sugar (RBS), thyroid-stimulating hormone (TSH), and serum calcium were sent for every subject. Other investigations, like fasting blood sugar (FBS), post-prandial blood sugar (PPBS), glycated hemoglobin (HbA1c), thyroid function test (TFT), serum creatine kinase (CK), serum parathyroid hormone (PTH), urinary Bence Jones protein, and X-ray or computed tomography (CT) scan of relevant site(s) were requested when indicated.

Patient with features suggestive of rheumatic disease were evaluated according to the established classification criteria (RA {2010 American College of Rheumatology (ACR)/European Alliance of Associations for Rheumatology (EULAR) classification criteria for rheumatoid arthritis} [14], SpA {Assessment in SpondyloArthritis international Society (ASAS) classification criteria for axial and peripheral spondyloarthritis} [15], SLE {2019 ACR/EULAR classification criteria for systemic lupus erythematosus} [16], and fibromyalgia {Widespread Pain Index (WPI) and Symptom Severity Score (SSS) for fibromyalgia} [17]). The case, fulfilling classification criteria for RA on the basis of joint involvement and duration of symptoms, was re-evaluated for the presence of SpA, SLE, or overlap. Subject with features suggestive of connective tissue disease (CTD) but not fulfilling classification criteria for any specific disease (e.g., SLE, systemic sclerosis {SSc}, Sjogren’s syndrome {SS}, or idiopathic inflammatory myositis {IIM}) was labeled as undifferentiated CTD (UCTD). Joint hypermobility syndrome (JHS) was diagnosed using the revised diagnostic criteria for the benign joint hypermobility syndrome [18,19]. Presence of chronic rheumatic disease in patients with JHS was mentioned as JHS/rheumatic disease overlap (e.g., JHS/RA overlap), otherwise BJHS. The investigations were performed in NMCTH.

Hypovitaminosis D in cases with rheumatic disease was termed hypovitaminosis D/rheumatic disease overlap (e.g., hypovitaminosis D/BJHS overlap). Rheumatic cause of widespread pain besides hypovitaminosis D was labeled as rheumatic disease for single diagnosis (e.g., BJHS) or rheumatic disease overlap for dual diagnoses (e.g., JHS/RA overlap).

Hypovitaminosis D was defined as a serum 25(OH)D level <30 ng/mL. Likewise, vitamin D insufficiency and deficiency were defined as serum 25(OH)D levels 20-30 ng/mL and <20 ng/mL, respectively (NMCTH laboratory uses the chemiluminescent immunoassay {CLIA} method, and has the same reference range) [7].

Details of the patients were recorded in a register. A pilot study was conducted on the initial 10% of the sample in our population, in accordance with their level of understanding, to validate the revised diagnostic criteria for benign joint hypermobility syndrome. Information was retrieved from the register and recorded in a self-constructed proforma that included demographic, clinical, and laboratory data (appendix 1). Bias was tried to overcome by following the above-mentioned process.

Data were entered and analyzed using Excel 2021 (Redmond, WA: Microsoft Corp.). Age was grouped according to Provisional Guidelines on Standard International Age Classifications (18-24, 25-44, 45-64, and 65+ years) [20]. The results were expressed as numbers, percentages, and mean±standard deviation (SD). Data analysis was done using Excel 2021. The prevalence of hypovitaminosis D was reported within 95% confidence interval. Graphical representations were shown using a pie chart, histogram, and line diagram.

## Results

**Figure 1:**
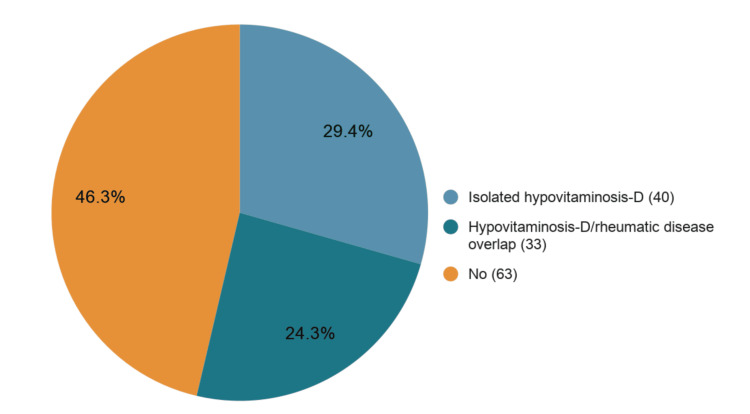
Hypovitaminosis D in adults with generalized aches (n=136).

A total of 136 adults presented to the rheumatology outpatient department (OPD) with generalized aches. There were 123 (90.4%) female and 13 (9.6%) male patients. Among them, 73 (53.7%) subjects had hypovitaminosis D (40 isolated and 33 with rheumatic disease overlap), with 66 (48.5%) women and seven (5.2%) men (Figure 1). There were 29 (21.3%) cases of vitamin D insufficiency and 44 (32.4%) cases of vitamin D deficiency (Table 1).

**Table 1:** Demographic characteristics of patients with hypovitaminosis D (n=73). n: total number of patients with hypovitaminosis D; Ins: insufficiency; Def: deficiency

Age group (years)	Isolated hypovitaminosis D				Hypovitaminosis D/rheumatic disease overlap			
	Female		Male		Female		Male	
	Ins	Def	Ins	Def	Ins	Def	Ins	Def
18-24	1	0	0	0	0	0	0	0
25-44	4	8	1	0	6	6	0	1
45-64	6	12	1	0	7	7	0	0
65 and above	0	5	1	1	1	3	1	1
Total	11	25	3	1	14	16	1	2

The mean age of subjects with hypovitaminosis D, including overlap, was 50.37±15.8 years (range: 18-89 years), and the mean value of vitamin D among them was 18.40±5.51 ng/mL (range: 6.9-29 ng/mL). The demographic characteristics of patients with hypovitaminosis D are shown in Table 1.

**Figure 2:**
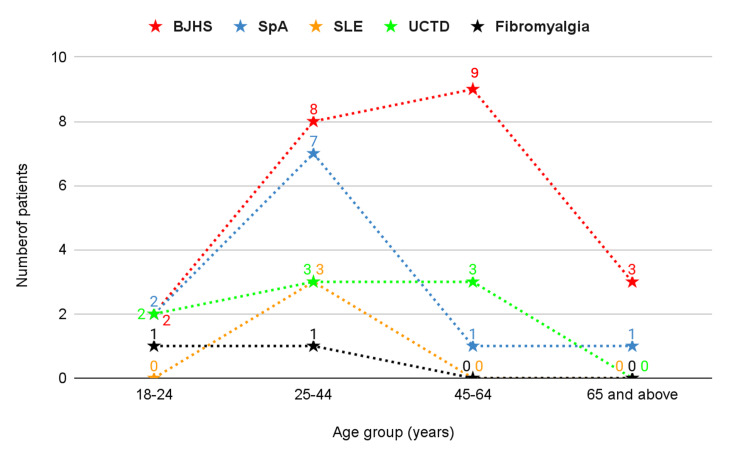
Single rheumatic cause of generalized aches besides hypovitaminosis D (n=47). n: total number of patients with a single rheumatic cause of generalized aches besides hypovitaminosis D; BJHS: benign joint hypermobility syndrome; SpA: spondyloarthropathy; SLE: systemic lupus erythematosus; UCTD: undifferentiated connective tissue disease

**Figure 3:**
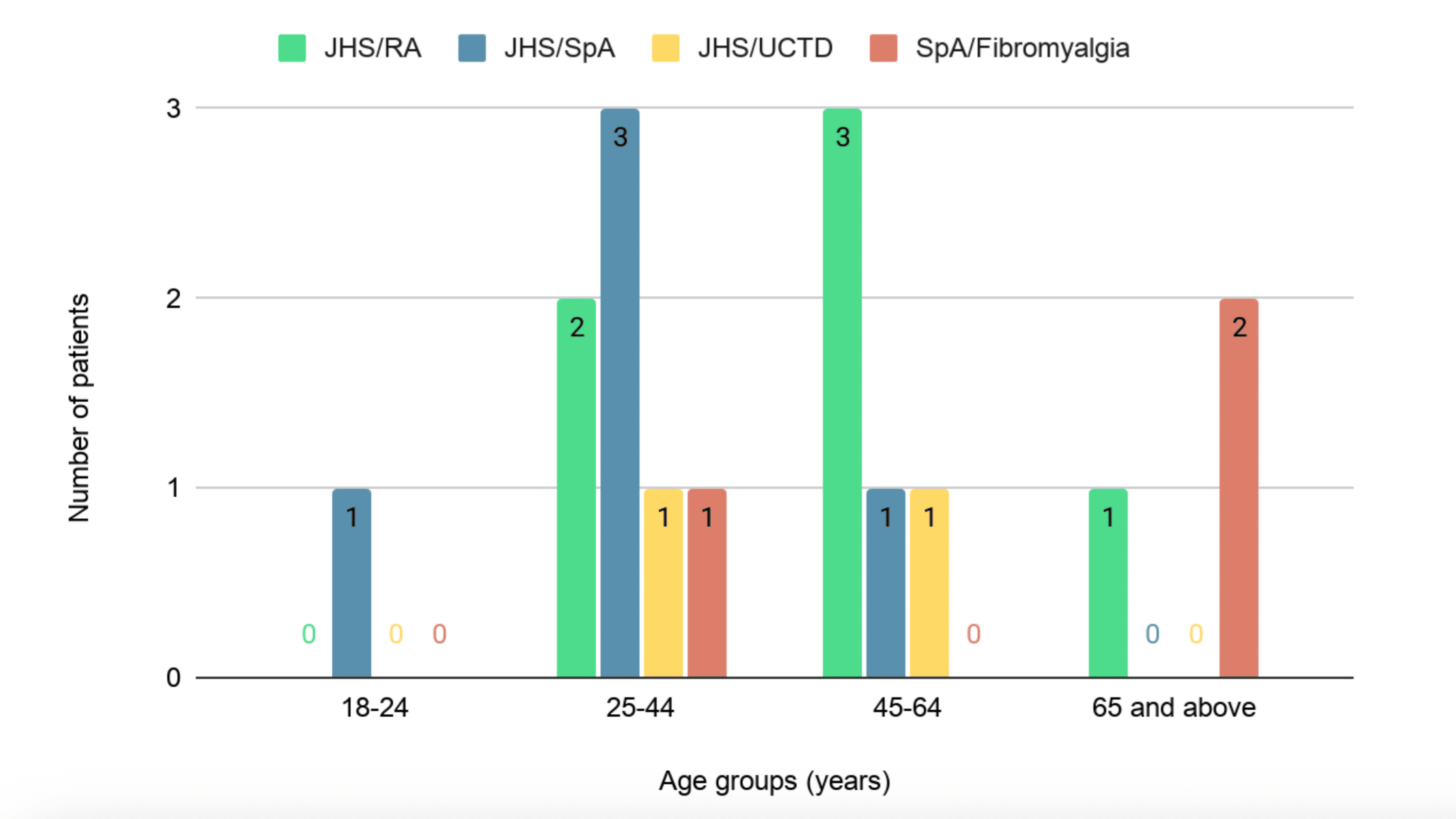
Rheumatic disease overlap as a cause of generalized aches besides hypovitaminosis D (n=16). n: total number of patients with rheumatic disease overlap as a cause of generalized aches besides hypovitaminosis D; JHS: joint hypermobility syndrome; RA: rheumatoid arthritis; SpA: spondyloarthropathy; UCTD: undifferentiated connective tissue disease

The causes of hypovitaminosis D/rheumatic disease overlap are shown in Table 2. Hypovitaminosis D/BJHS was found to be the most common among overlaps. The remaining 63 (46.3%) cases had only rheumatic disease as the cause of generalized aches (47 single diagnoses and 16 overlap diagnoses), with 57 female and six male subjects (Figures 2, 3). The most common cause of widespread pain besides hypovitaminosis D was BJHS. We did not find any cases of infection, diabetes mellitus, or thyroid disorders as causes of generalized aches.

**Table 2:** Hypovitaminosis D/rheumatic disease overlap (n=33). n: total number of patients with hypovitaminosis D/rheumatic disease overlap; BJHS: benign joint hypermobility syndrome; RA: rheumatoid arthritis; SpA: spondyloarthropathy; SLE: systemic lupus erythematosus

Hypovitaminosis D/rheumatic disease overlap	Number of patients
Hypovitaminosis D/BJHS overlap	20
Hypovitaminosis D/RA overlap	4
Hypovitaminosis D/SpA overlap	5
Hypovitaminosis D/SLE overlap	3
Hypovitaminosis D/fibromyalgia overlap	1
Total	33

## Discussion

Among various etiologies of generalized aches, hypovitaminosis D is one of the most common causes. In our country, we have been observing supplementation of oral vitamin D casually by attending physicians, paramedics, or over the counter (OTC), leading to inadequate data on hypovitaminosis D and neglecting other sources of widespread pain. Therefore, diligent consideration of various causes of widespread pain during assessment is crucial.

In our study, hypovitaminosis D was found in 53.7% of adults with generalized aches. One study from Nepal showed that 82.6% of the population with generalized aches, including 25.7% fibromyalgia overlap, had hypovitaminosis D [13]. Siddiqee et al. in 2021 observed vitamin D deficiency in 68% of individuals across five South Asian countries, with significant heterogeneity. They reported low vitamin D levels in 57% of Nepalese people [9]. Shah et al. in 2021 found that 57.7% of patients had low vitamin D levels [21]. Other publications from Nepal have reported frequencies of hypovitaminosis D ranging from 70.7% to 84.5% [10-12,22]. The low prevalence of hypovitaminosis D in the present study may be due to the enrollment criteria and small sample size. In addition, the increasing culture of taking OTC oral vitamin D supplements in recent years may have led to decreased frequency of hypovitaminosis D.

We found insufficiency and deficiency of vitamin D in 21.3% and 32.4% of subjects, respectively. Studies done in different parts of Nepal have shown variable frequency of vitamin D insufficiency as well as deficiency (insufficiency: 35.9% [11], 42.6% [22], 42.8% [21], 74.1% [10], and 92% [12]; deficiency: 8% [12], 14.9% [21], 25.9% [10], 34.8% [11], 57.4% [22], and 62.6% [13]). This wide variation across publications may be due to differences in research settings and selection bias.

The mean vitamin D level in patients with hypovitaminosis D, including overlap, was 18.40 ng/mL (range: 6.9-28.2 ng/mL). In contrast to our study, the mean value of low levels of vitamin D is uncommon in other literature. This has led to a lack of data on the average level of vitamin D among subjects with hypovitaminosis D in our country.

The mean age of subjects with hypovitaminosis D was 50.37 years (range: 18-89 years), with female predominance (48.5%). Likewise, Tamang et al. in 2023 and Tiwari et al. in 2024 observed mean ages of 45.35 years and 43.25 years, respectively, similar to ours [12,22]. Several studies in Nepal have found that low levels of vitamin D were more prevalent in women; 37.3% [7], 39.7% [21], 66.2% [13], 70.5% [22], 70.8% [12], 71.4% [10], and 72.4% [11]. This might be attributed to their time spent indoors.

In the present study, we also observed rheumatic disease overlap with low levels of vitamin D. Publications on rheumatic disease overlapping hypovitaminosis D are rare, though Vaidya et al. in 2014 have shown hypovitaminosis D/fibromyalgia overlap [13]. In our study, we found a significant number of rheumatic diseases to be an isolated cause of generalized aches, similar to that reported by Reilly in 1999 [2]. This study revealed rheumatic disease overlap, a unique cause of widespread pain.

The prevalence of hypovitaminosis D varies across the published literature. This may be due to differences in study design and a lack of uniformity in laboratory values used to indicate vitamin D insufficiency and deficiency. The present study observed only rheumatic disease, including overlap, though other non-rheumatic causes are also responsible for generalized aches. This demands further study on every possible cause(s) of global musculoskeletal pain, which is common among adults and frequently missed in clinical settings.

Strengths of the study

This study, which aimed to determine the prevalence of hypovitaminosis D in adults with generalized aches, is a novel work conducted in Nepal. Publications on other causes of widespread pain, including overlap, from our country are also rare. Our study can help in the early diagnosis of these overlooked causes of generalized aches in resource-constrained settings. This study can also be used to calculate the sample size for hypovitaminosis D in adult patients with widespread pain.

Limitations of the study

The present study has several limitations. First of all, it is an observational study done in the rheumatology OPD of a tertiary care hospital. So, our study may not cover all cases of generalized aches in the community. Our center provides only two rheumatology OPD services per week, which may influence the number of patients. Cases <18 years of age were not included. The body mass index (BMI) of subjects was not recorded. The patient's diet and duration of sun exposure were not assessed. Conditions that may cause hypovitaminosis D were not evaluated. Joint hypermobility syndrome was diagnosed only on the basis of clinical evaluation. Lastly, the retrospective nature of the study might have influenced the data available for research.

## Conclusions

Hypovitaminosis D is a common cause of widespread pain in adults. Other causes, especially rheumatic disease, are also responsible for this condition. Among them, BJHS is the most common. Even rheumatic diseases can overlap with low levels of vitamin D to cause global musculoskeletal pain. Generalized aches may be due to rheumatic disease overlap in the absence of hypovitaminosis D. So, meticulous history and examination are the key to approach.

## Appendices

Appendix 1

Name:

Age/sex:

**Table 3:** Proforma: prevalence of hypovitaminosis D in adults with generalized aches.

Variables	Results
1. Level of 25(OH)D	1. Low. 2. Normal
If low	1. 20-30 ng/mL (insufficiency) 2. <20 ng/mL (deficiency)
2. Complete blood count (CBC)	1. Normal. 2. Abnormal
If abnormal, then mention the value	
3. Erythrocyte sedimentation rate (ESR)	1. Low. 2. Normal
4. C-reactive protein (CRP)	1. Low. 2. Normal
5. Random blood sugar (RBS)	1. Low. 2. Normal. 3. High
6. Thyroid-stimulating hormone (TSH)	1. Low. 2. Normal. 3. High
7. Serum calcium	1. Low. 2. Normal. 3. High
8. 2010 ACR/EULAR classification criteria for rheumatoid arthritis	1. Fulfill. 2. Does not fulfill
9. ASAS classification criteria for axial and peripheral spondyloarthritis (SpA)	1. Fulfill. 2. Does not fulfill
10. 2019 EULAR/ACR classification criteria for systemic lupus erythematosus	1. Fulfill. 2. Does not fulfill
11. Widespread Pain Index (WPI) and Symptom Severity Score (SSS) for fibromyalgia	1. Fulfill. 2. Does not fulfill
12. Revised diagnostic criteria for the benign joint hypermobility syndrome	1. Fulfill. 2. Does not fulfill
If needed	
13. Fasting blood sugar (FBS)	1. Low. 2. Normal. 3. High
14. Post-prandial blood sugar (PPBS)	1. Low. 2. Normal. 3. High
15. Glycated hemoglobin (HbA1C)	1. Normal. 2. High
16. Thyroid function test (TFT)	1. Normal. 2. Abnormal
If abnormal, then mention the value	
17. Serum creatinine kinase (CK)	1. Low. 2. Normal. 3. High
18. Serum parathyroid hormone (PTH)	1. Low. 2. Normal. 3. High
19. Urinary Bence Jones protein	1. Present. 2. Absent
20. X-ray/computed tomography (CT) scan of relevant site(s)	1. Suggestive. 2. Not suggestive
